# Difference and Cluster Analysis on the Carbon Dioxide Emissions in China During COVID-19 Lockdown via a Complex Network Model

**DOI:** 10.3389/fpsyg.2021.795142

**Published:** 2022-01-12

**Authors:** Jun Hu, Junhua Chen, Peican Zhu, Shuya Hao, Maoze Wang, Huijia Li, Na Liu

**Affiliations:** ^1^School of Economics and Management, Fuzhou University, Fuzhou, China; ^2^School of Management Science and Engineering, Central University of Finance and Economics, Beijing, China; ^3^School of Artificial Intelligence, Optics and Electronics, Northwestern Polytechnical University, Xi'an, China; ^4^School of Science, Beijing Post and Telecommunications University, Beijing, China

**Keywords:** time series, visibility graph, complex network, carbon dioxide emissions, K-means

## Abstract

The continuous increase of carbon emissions is a serious challenge all over the world, and many countries are striving to solve this problem. Since 2020, a widespread lockdown in the country to prevent the spread of COVID-19 escalated, severely restricting the movement of people and unnecessary economic activities, which unexpectedly reduced carbon emissions. This paper aims to analyze the carbon emissions data of 30 provinces in the 2020 and provide references for reducing emissions with epidemic lockdown measures. Based on the method of time series visualization, we transform the time series data into complex networks to find out the hidden information in these data. We found that the lockdown would bring about a short-term decrease in carbon emissions, and most provinces have a short time point of impact, which is closely related to the level of economic development and industrial structure. The current results provide some insights into the evolution of carbon emissions under COVID-19 blockade measures and valuable insights into energy conservation and response to the energy crisis in the post-epidemic era.

## 1. Introduction

Since the end of 2019, the outbreak of COVID-19 has had a major impact on the health and property of people all over the world. The world is almost in a state of "general lockdown" state as many governments have taken "shock" measures to prevent the outbreak by shutting down work and schools, closing borders, restricting travel and social isolation (Badesa et al., 2021). Despite the enormous losses caused by the lockdown, it is extremely important in controlling the epidemic, reducing carbon dioxide emissions, and prohibiting damage to people's health (Cicala et al., 2021). Relevant studies revealed that the lockdown during the COVID-19 resulted in significant reductions in stated emissions of nitrogen dioxide NO_2_ and carbon dioxide CO_2_ due to reduced transport activities (Celik and Gul, 2021), demand for electricity and the cessation of industrial activity, massive reductions in human activity, and significant reductions in fossil energy consumption (Usman et al., 2021). In China, for example, annual carbon dioxide emissions in 2020 was 2% lower than those in 2019, while this is the first decline since 1997 (Liu et al., 2020b). This situation calls for humanity's attention to the importance of protecting the ecological environment and realizing the balanced development of economy and ecology (Hanna et al., 2020). Under this background, studies on the transition to a low-carbon economy are increasingly prominent (Ionescu, 2021b).

The low carbon economy is important for environmental performance, sustainable energy and promoting green financial behavior (Ionescu, 2020a), carbon pricing and pollution taxes continue to prove to be cost-effective economic instruments to curb greenhouse gas emissions (Ionescu, 2019, 2020b). Pflugmann and De Blasio (2020)'s research expands the scope of sustainable energy research through the new energy perspective of renewable hydrogen (Pflugmann and De Blasio, 2020). Ionescu (2021) confirmed the development and evolution of green finance in the COVID-19 pandemic for low-carbon energy, sustainable economic development and climate change mitigation by the data from NGFS and the United Nations (Ionescu, 2021a). In this study, we focus on investigating carbon emissions in China in 2020, which is under the global prevalence of COVID-19, because China is one of the major global emitters of greenhouse gases and the first country to take control measures against the COVID-19 (Liu et al., 2021c). Moreover, the COVID-19 had a significant impact on greenhouse gas emissions and the reduction of atmospheric pollutants in China (Filonchyk et al., 2020; Liu et al., 2020b). Zheng, et al. (2020) confirmed that China's CO2 emissions reduced by 11.5% from January to April 2020 compared to the amount during the same period in 2019 (Zheng et al., 2020). Exploring the impact of COVID-19 on China's carbon emissions is of great significance for promoting a low-carbon economy globally (Liu et al., 2020b).

However, there is a lack of real-time, quantitative studies on carbon emissions under COVID-19 combined with lockdown policies (Sreekanth et al., 2021; Zhou et al., 2021). In existing studies on COVID-19 and carbon emissions, carbon emission data are often collected monthly or annually, making the time lag. The complex network is a method to analyze the characteristics of relevant data from a temporal perspective and present them through graphs, animations, etc. (Lacasa et al., 2008). This method allows for a dynamic presentation of carbon emissions over time and deeply reveal the information behind the data (Chimmula and Zhang, 2020). For example, Wang, et al. (2021) used complex network theory to construct a network of carbon emission flows in key sectors in China across industries (Wang et al., 2021b). Similarly, to investigate the impact of industry-driven effects on carbon emissions, Zheng et al. (2021) combined multi-regional input-output theory with complex network theory to construct a network of industry-driven effects, the study showed that the different effects of industry-driven effects had a positive impact on carbon emissions (Zheng et al., 2021). However, previous studies have generally assumed regions to be homogeneous and little attention has been paid to differences in economic development when analyzing the greenhouse gas emissions. While carbon emissions are closely related to economic development level, regional transport and industry conditions, there is a lack of studies on regional heterogeneity. Therefore, there is an urgent need for real-time, high spatial emission datasets to quantify the impact of COVID-19 on carbon emissions across China.

The contributions of this paper are as follows: First, in terms of research methodology, the complex network method compensates for the data shortcomings of previous studies on lagging carbon emissions and extends the application of complex network analysis methods. Secondly, in practical application, the paper takes China, where the lockdown measures are stringent and carbon emissions have a large impact, and combines the dynamic change process of carbon emissions during the COVID-19 pandemic with the lockdown policies to provide scientific suggestions for the development of a low carbon economy in the post-epidemic era.

## 2. Methodology and Data

### 2.1. Visibility Graph

Network topology can be used to indicate the current status and behavior of system which may not be shown in the time series explicitly (Fan et al., 2019). In Lacasa et al. (2008) found a new algorithm to map time series into complex networks, which is a key problem in the research on time series, namely, visibility graph (*VG*). VG has also been proved to have two particularly eminent advantages: its topology inherits the characteristics of the time series involved and it shows Supplementary Material through degree distribution. Moreover, this novel relation between the time series and complex networks can provide a great many possibilities for the study of complex signals (Hu et al., 2020).

**Figure 1:**
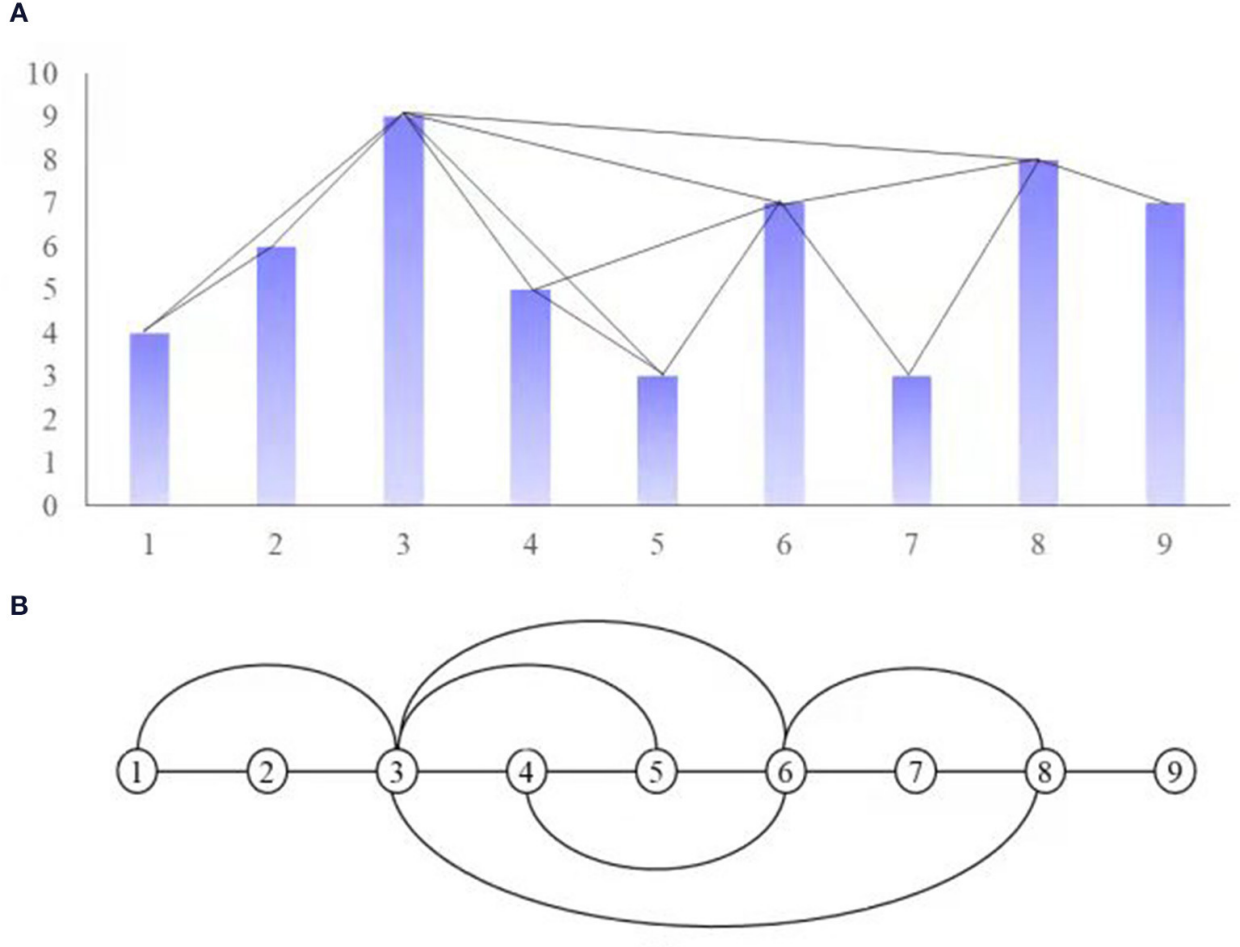
Visiblity graph.

The algorithm of the network building method follows the principle: each node in the network corresponds to each time point in the discrete time-series data. Connect the two points in these nodes that meet the prescribed visibility rules in advance, which forms the edge of the network. The basic idea of the algorithm is shown in Figure 1. In the upper part of the graph, 18 straight squares are used to represent the first 20 data points in a periodic time series (Figure 1A). The height of the straight squares in the subgraph (Figure 1A) represents the data value of each time point. If the top of the two straight squares is visible to each other, the corresponding two points are connected in the network in the subgraph (Figure 1B). In graph (Figure 1B), each real point corresponds to the data point in the time series one by one. Specifically, each node can not be connected to itself, and each visible line can not pass through any straight bars.

If there are any two points of the carbon emission (*t*_*a*_,*x*_*a*_), and (*t*_*b*_,*x*_*b*_), the every points (*t*_*c*_,*x*_*c*_) between these two points, safeties the Equation (1), there will be an edge to link these two points,


(1) $$\begin{array}{l} x_{b}<x_{a}+\left(x_{c}-x_{a}\right)\frac{t_{b}-t_{a}}{t_{c}-t_{a}}. \end{array}$$

### 2.2. Complex Network

The study of complex networks is permeating many different fields from mathematical and scientific disciplines to life and engineering disciplines, and the scientific understanding of the quantitative and qualitative characteristics of complex networks has become an extremely important and challenging topic. The advantage is that the structure of complex systems and the relationship between structure and function can be explored (Qiao et al., 2021). Network is a graph composed of nodes and edges. Its mathematical expression is *G* = (*N, V*), where *N* is the aggregate of nodes and *V* is the set of edges. Complex network is a useful tool to analyze social problems, management science, computer, statistics and other problems. In this study, by transforming the time series into a network, we analyze the time series data through the statistical characteristics of the complex network, and mine more useful information in the time series, so that we can get more accurate analysis results. The statistical characteristics of complex networks are as follows:

#### 2.2.1. Degree

In the complex network, *d*_*i*_ endnotes degree of the node *i*, which is the the number of the edges connected to the node *i*, and the average degree < *K* > is the the average value of all the nodes' degree,


(2) $$\begin{array}{l} <k>=\frac{1}{N}\overset{N}{\underset{i=1}{\sum }}d_{i}, \end{array}$$

where *N* is the number of the nodes in this network.

The degree distribution *p*(*k*) is the frequencies of nodes with the *d*_*i*_ = *k* in the complex network.

#### 2.2.2. Average Path Length and Diameter

In the network science, the network characteristics can be used to describe by the distance between any two nodes, e.g., average path length and diameter. It's easy to know that the short path of the node *i* and node *j* is *d*_*ij*_, and the average path length is the average short path *L* of all the short path between any two nodes, as given blow:


(3) $$\begin{array}{l} L=\frac{1}{N\left(N-1\right)}\underset{1\leq i<j\leq N}{\sum }d_{ij}, \end{array}$$

and the diameter is the longest path in the all short path.

#### 2.2.3. Clustering Coefficient

Cluster coefficient is a index to describe the aggregation degree of the each nodes in the network, and the average cluster coefficient endnote the the aggregation degree of the network. The definition of the cluster coefficient:


(4) $$\begin{array}{l} C_{i}=\frac{E_{i}}{C_{k_{i}}^{2}}, \end{array}$$

where *k*_*i*_ is the number of neighbors of the node *i*, *E*_*i*_ is the number of actually existing edges, $C_{k_{i}}^{2}$ is the number of theoretically existing edges. And average cluster coefficient is:


(5) $$\begin{array}{l} C=\frac{1}{N}\overset{N}{\underset{i}{\sum }}C_{i}, \end{array}$$

where *C*_*i*_ is the cluster coefficient of node *i*, *N* is the number of nodes.

### 2.3. K-means

A clustering algorithm is a method for automatically dividing a set of unlabeled data into multiple classes, which is an unsupervised learning method (Banerjee et al., 2005), aiming to categorize similar data into certain clusters (Tang et al., 2018). K-means clustering is the clustering method of assignments of data points based on similarity, and it's been proven to be more effective to optimize results (Yan et al., 2020). By manually setting the parameter *K*, *n* objects can be divided into *k* categories, which makes the similarity within the same category the highest, while the similarity between categories the lowest.

Based on a given set of *N* objects, the *K*-means algorithm will build *k* methods partition clustering methods, and each partition clustering is a cluster. The data is divided into n clusters, each cluster has at least one data object, and each data object must belong to and can only belong to one cluster. At the same time, the similarity of data objects in the same cluster is high, and the similarity of data objects in different clusters is low. The clustering similarity is calculated using the average value of objects in each cluster.

The processing flow of the *K*-means algorithm is as follows. First, it randomly selects *k* data objects, each data object represents a cluster center, namely, select *k* initial centers. Second, for each remaining object, it assigns an object to the most similar cluster according to the similarity (distance) with each cluster center. Then, it recalculates the average value of all objects in each cluster as the new cluster center. This process is repeated until the criterion function converges, as the cluster center does not change significantly. The mean square error is usually used as the criterion function to minimize the sum of the squares of the distances from each point to the nearest cluster center.

**Figure 2:**
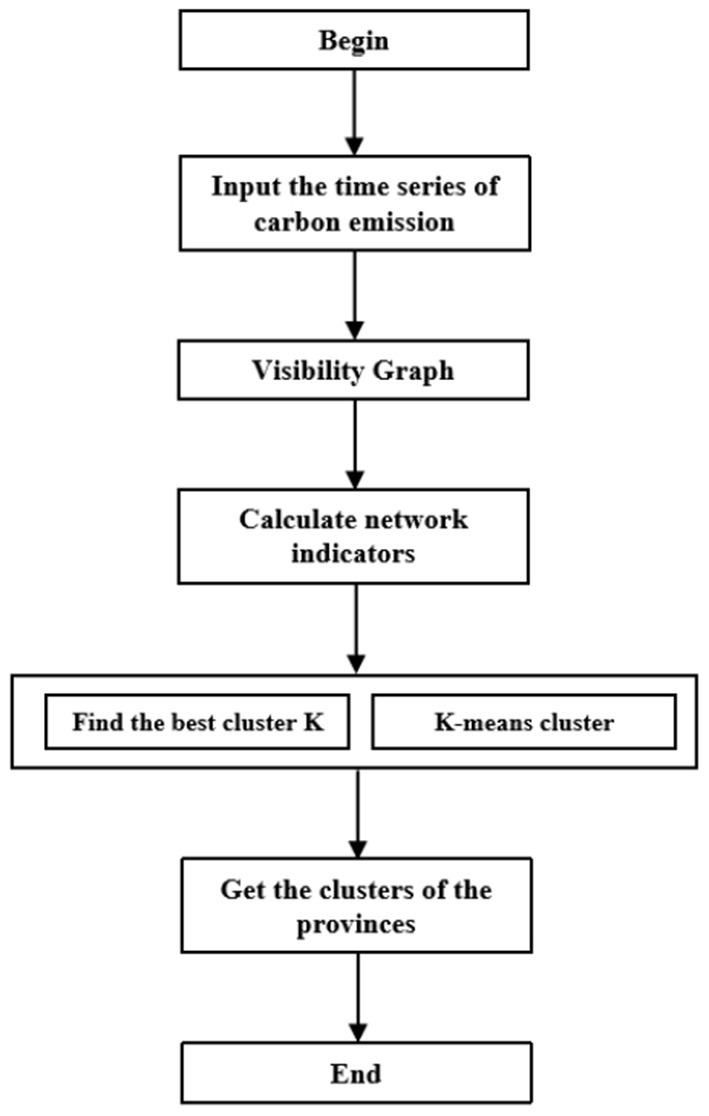
Flow chart of research on carbon dioxide emissions in China.

The technical route of this paper is illustrated in Figure 2. Firstly, in order to explore the nodes and linkage system of carbon emissions during the COVID-19 epidemic, time series analysis and visualization of carbon emissions are made. Secondly, a complex network of indicators is constructed to further explore the power-law distribution of nodes. Following this, a K-means analysis is conducted to incorporate regional heterogeneity and to form the final clusters.

### 2.4. Data Source

We collected daily data of daily confirmed COVID-19 cases of 30 provinces in China during 2020 from Git Hub. The macro indicators such as per capita GDP, secondary industry growth index and passenger volume of each province in 2020 were obtained from the National Bureau of Statistics and local statistical yearbooks. In addition, China's carbon emissions data of 30 provinces in 2020 are from the website (https://www.carbonmonitor.org.cn/) and reference (Liu et al., 2020a).

## 3. Result

### 3.1. Descriptive Analysis

**Figure 3:**
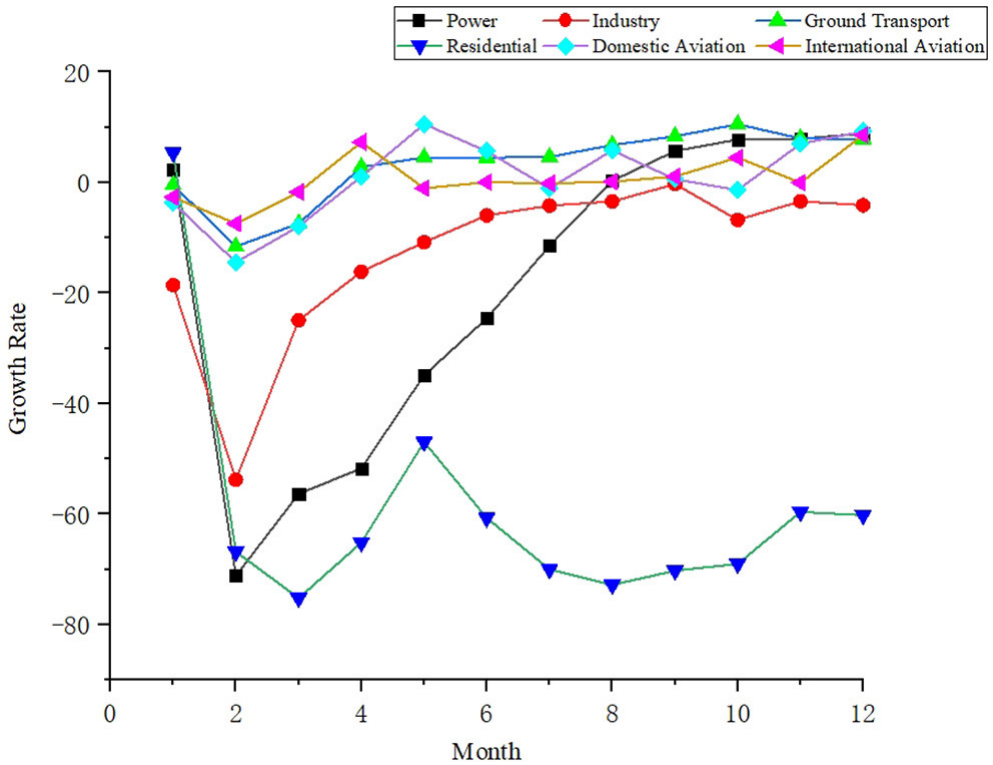
Trend line for the growth rate of carbon emissions of 30 provinces in 2020.

We analyzed the total carbon emissions of 30 provinces in 2020. Table 1 shows the statistical characteristics and changing trend of the total emissions of each province. We can see that the provinces with high carbon emissions are Shandong, Guangdong and Jiangsu. Figure 3 shows the growth rate trend of carbon emissions by sector in 2020. Statistically, we can find that COVID-19 has brought about a short-term decrease in carbon emissions, especially during the period of national lockdown from January to March 2020, followed by a gradual increase in industrial, energy and residential carbon emissions. The impact on the transport sector is huge, because not only are carbon emissions from the lockdown down, but all transport emissions were in negative growth until September. The international aviation emissions remained in negative growth, due to the global COVID-19 pandemic, which greatly reduced international air transport.

**Table 1:** Statistical indicators of carbon dioxide emissions of 30 provinces.

	**AVG**	**SE**	**Med**	**SD**	**Var**	**Kur**	**Ske**	**Min**	**Max**	**Sum** ^***a***^
Beijing	0.16	0.00	0.16	0.04	0.002	–0.45	–0.06	0.07	0.26	60.25
Tianjin	0.22	0.00	0.22	0.06	0.004	1.16	–1.12	0.06	0.32	79.37
Hebei	1.31	0.01	1.38	0.27	0.074	–0.86	–0.63	0.75	1.83	478.76
Shanxi	0.97	0.01	1.02	0.23	0.053	0.18	–1.03	0.39	1.39	353.62
Inner Mongolia	1.26	0.02	1.34	0.35	0.123	1.23	–1.25	0.31	1.76	459.81
Liaoning	0.65	0.01	0.69	0.16	0.026	–1.06	–0.46	0.34	0.95	239.45
Jilin	0.30	0.00	0.30	0.08	0.006	–1.15	–0.05	0.16	0.43	108.90
Heilongjiang	0.35	0.00	0.34	0.09	0.008	–1.12	–0.09	0.19	0.51	126.76
Shanghai	0.24	0.00	0.23	0.07	0.006	0.56	–0.39	0.06	0.37	86.30
Jiangsu	1.88	0.02	1.95	0.44	0.193	–0.43	–0.47	0.91	2.64	687.49
Zhejiang	1.33	0.02	1.43	0.32	0.101	–0.63	–0.73	0.65	1.91	485.67
Anhui	1.33	0.01	1.35	0.26	0.070	–0.44	–0.21	0.77	1.87	488.10
Fujian	0.88	0.01	0.88	0.18	0.031	–0.98	–0.33	0.51	1.19	323.08
Jiangxi	0.79	0.01	0.79	0.16	0.025	–0.97	–0.10	0.48	1.14	287.75
Shandong	2.06	0.02	2.19	0.42	0.176	0.10	–0.98	1.03	2.91	753.34
Henan	1.25	0.01	1.33	0.23	0.053	–0.29	–0.98	0.72	1.60	458.76
Hubei	0.82	0.01	0.82	0.25	0.060	–0.41	–0.02	0.32	1.32	301.83
Hunan	0.81	0.01	0.81	0.16	0.025	–0.24	0.38	0.51	1.16	296.21
Guangdong	1.82	0.02	1.93	0.40	0.164	–0.74	–0.51	0.97	2.55	666.28
Guangxi	0.90	0.01	0.89	0.15	0.024	–0.40	0.25	0.58	1.24	328.07
Hainan	0.15	0.00	0.15	0.03	0.001	–1.01	–0.32	0.09	0.19	53.62
Chongqing	0.48	0.00	0.49	0.09	0.008	–0.55	0.27	0.28	0.68	176.79
Sichuan	0.95	0.01	0.94	0.15	0.023	–0.60	0.33	0.57	1.27	347.17
Guizhou	0.87	0.01	0.90	0.20	0.041	–0.68	–0.12	0.46	1.32	319.63
Yunnan	0.80	0.01	0.83	0.14	0.019	–0.72	–0.11	0.47	1.08	293.52
Shaanxi	0.83	0.01	0.88	0.15	0.023	0.34	–1.12	0.43	1.16	304.00
Gansu	0.44	0.00	0.47	0.09	0.007	–0.19	–0.85	0.24	0.63	162.45
Qinghai	0.09	0.00	0.10	0.02	0.000	0.49	–1.08	0.04	0.13	33.22
Ningxia	0.45	0.01	0.48	0.11	0.013	1.41	–1.44	0.13	0.63	162.99
Xinjiang	0.93	0.01	1.00	0.23	0.052	1.46	–1.41	0.29	1.30	340.60

a *AVG, Average value; SE, Standard error; Med, Median; SD, Standard deviation; Var, Variance; Kur, Kurtosis;, Ske, Skewness; Min, Minimum; Max, Maximum*.

In the case of confirmed COVID-19 in China, the country is the first to fight the outbreak compared to other countries in the epidemic area. Existing studies usually divide the time period into seed period from 2020.1.1 to 2020.1.23, and lockdown measure management from 2020.1.24 to 2020.3.8 (Liu et al., 2021a). At the time of the Wuhan outbreak in January, there was no clear evidence of direct differentiation from other known influenza viruses. During the first days of the transmission, local authorities were busy controlling the spread of the disease and ensuring that all COVID-19 patients were well cared for. Meanwhile, infected areas such as the Wuhan seafood market were immediately closed and thoroughly disinfected. With Hubei province declaring a level II public health emergency response, Wuhan closed its access on January 23, then Hubei and other provinces across the country began to collective fight the virus. As a result of this series of "closures and social distancing" policies, the number of confirmed cases in China has dropped by 71 percent (Thu et al., 2020). After March, China has been cautiously and gradually reopening its economy, and there have been few large-scale infections as the country has stepped up measures to ensure national security. The epidemic has been managed with various but effective actions by different provinces, all of which have succeeded in adopting enthusiastic and cooperative people and appropriate government measures (Balsalobre-Lorente et al., 2020). Table 2 shows China's lockdown measures during this period.

**Table 2:** China's lockdown measures.

**Lockdown**	**Area**	**Requirement**	**Means**
City	Cities with multiple confirmed cases, such as Hubei	Restrictions on the mobility of people, suspend all work, business and schools	Suspension and closure of all modes of transport, restrictions on private cars, community closures
Road	National and provincial highways, entrances and exits, and some roads in the city	Temporary closure of highway department exits and vehicles were prohibited	Warning signs, cordons, roadblocks, etc.
Community	There were no or few confirmed cases, such as Hebei.	Closed management; no foreign vehicles; no foreigners; restricted access to residents	Only one passageway is reserved for residents with access cards and limits the number of outside times
Door	Returning residents of infected areas or those close with confirmed cases	Forced isolation for more than 14 days and strictly forbidden to go outside	Real-time reporting of body temperature and other health conditions through in-home testing

*From: China's provinces, cities, and community announcement*.

### 3.2. Carbon Emissions and Lockdown

**Figure 4:**
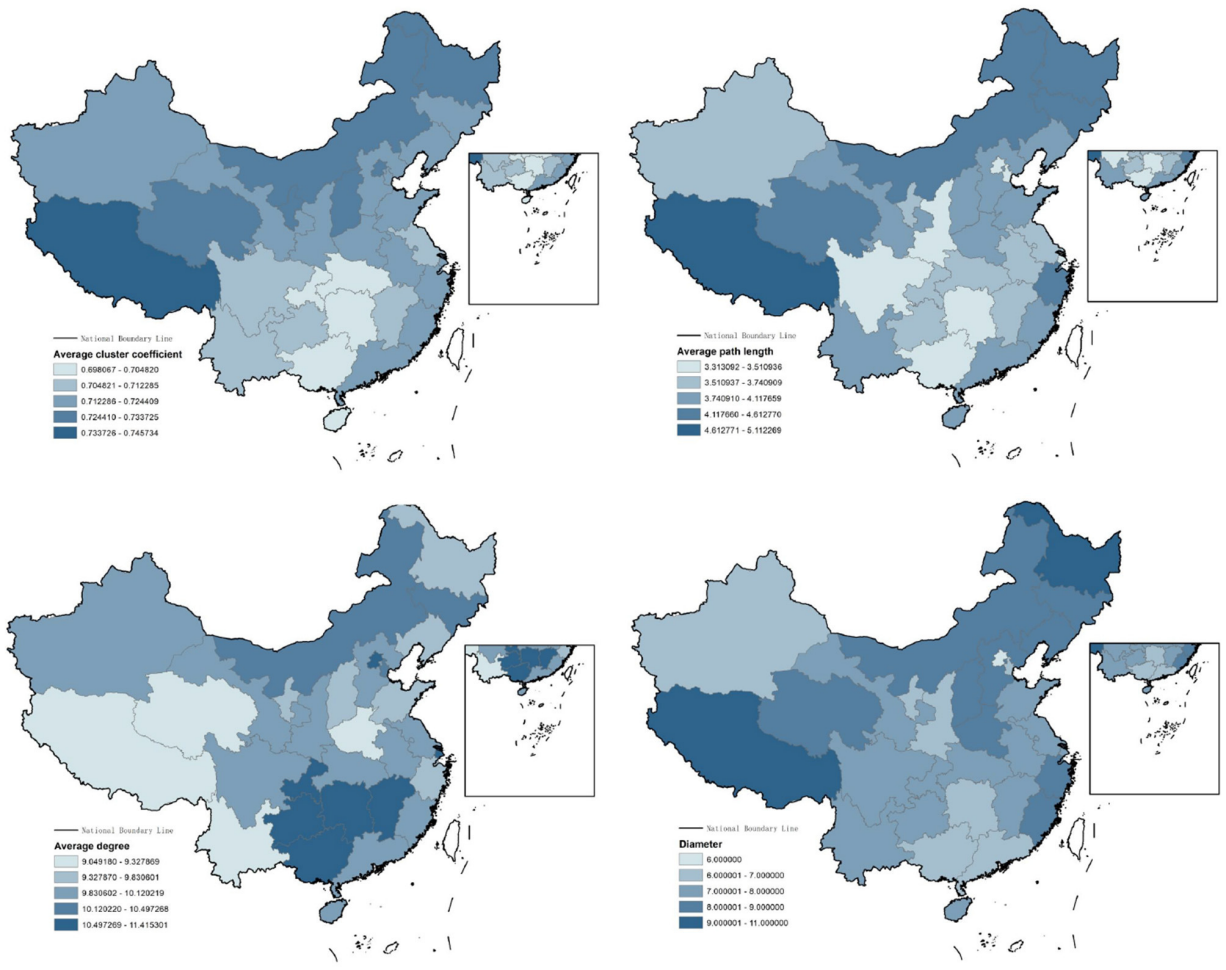
Network characteristics of 30 provinces analyzed through the visibility graph.

Figure 4 shows the network characteristics of 30 provinces analyzed through the visibility graph, where the darker the color, the greater the value of the topology measures of this network. From the results, we conclude that: (1) The network topology can indicate and inherit the properties of the current network. (2) Networks implemented in the corresponding time series can reveal the true nature of the time series in question with reference to specified indicators. (3) the link between complex networks and time series provides researchers with a general method to examine the process of complex signals with several different possibilities.

**Figure 5:**
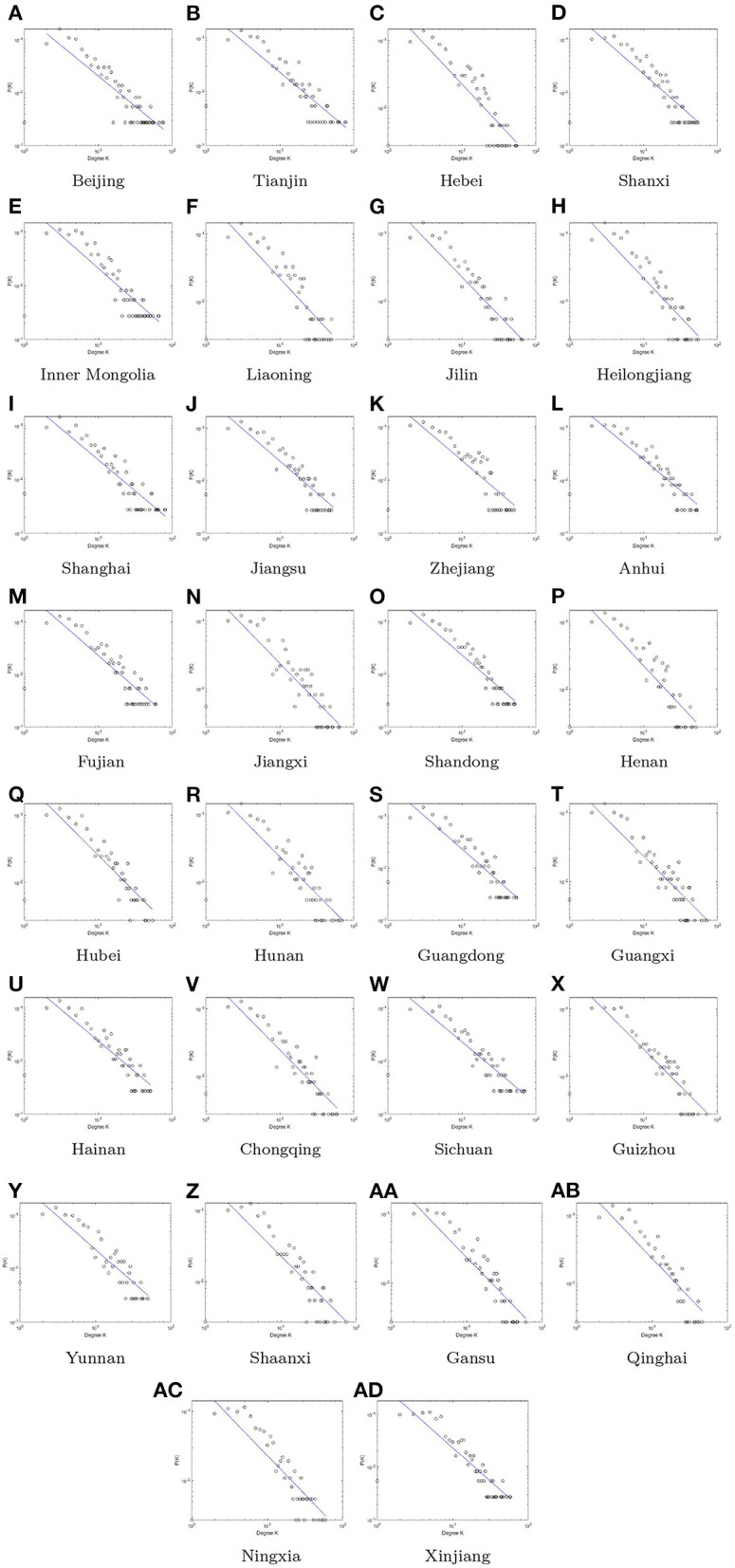
The degree distribution of different provinces in China. **(A)** Beijing, **(B)** Tianjin, **(C)** Hebei, **(D)** Shanxi, **(E)** Inner Mongolia, **(F)** Liaoning, **(G)** Jilin, **(H)** Heilongjiang, **(I)** Shanghai, **(J)** Jiangsu, **(K)** Zhejiang, **(L)** Anhui, **(M)** Fujian, **(N)** Jiangxi, **(O)** Shandong, **(P)** Henan, **(Q)** Hubei, **(R)** Hunan, **(S)** Guangdong, **(T)** Guangxi, **(U)** Hainan, **(V)** Chongqing, **(W)** Sichuan, **(X)** Guizhou, **(Y)** Yunnan, **(Z)** Shaanxi, **(AA)** Gansu, **(AB)** Qinghai, **(AC)** Ningxia, and **(AD)** Xinjiang.

Figure 5 shows the degree distribution of visibility graphs of 30 provinces in China, from the sub-figures, it's easy to find that the degree distribution of 30 provinces are long-term correlation and the power law distribution, which means that the VG of these 30 provinces are the scale-free network, and the distribution fitting parameters and the goodness of fit R2 of 30 provinces are shown in Table 3. According to the visual rules and the degree distribution we know that only few with large degree, which means that few time have the large influence. The majority node with low degree, which is that the influence range of the most time nodes is short. This also reflects to some extent the timeliness of the epidemic prevention and control policy.

**Table 3:** The fitting parameters *ax*^−*b*^ and goodness of fit *R*^2^ of degree distribution of 30 provinces.

	**a**	**b**	***R* ^2^**
Beijing	0.290	1.238	0.614
Tianjin	0.327	1.118	0.659
Hebei	0.336	1.091	0.548
Shanxin	0.378	0.974	0.581
Inner Mongolia	0.349	1.052	0.614
Liaoning	0.337	1.086	0.530
Jilin	0.309	1.176	0.580
Heilongjiang	0.323	1.130	0.550
Shanghai	0.329	1.110	0.676
Jiangsu	0.391	0.940	0.619
Zhejiang	0.357	1.029	0.527
Anhui	0.364	1.009	0.636
Fujian	0.396	0.927	0.637
Jiangxi	0.315	1.156	0.619
Shandong	0.384	0.956	0.595
Henan	0.343	1.071	0.525
Hubei	0.320	1.138	0.610
Hunan	0.302	1.198	0.630
Guangdong	0.411	0.888	0.645
Guangxi	0.283	1.263	0.636
Hainan	0.363	1.014	0.627
Chongqing	0.321	1.137	0.631
Sichuan	0.344	1.068	0.665
Guizhou	0.323	1.130	0.631
Yunnan	0.403	0.909	0.621
Shaanxi	0.268	1.317	0.527
Gansu	0.318	1.146	0.556
Qinghai	0.339	1.082	0.523
Ningxia	0.308	1.177	0.558
Xinjiang	0.432	0.840	0.680

In the short term, from January to March 2020, with the emergence of patients with unexplained pneumonia in Wuhan, the country successively from the beginning of the implementation of lockdown measures. At this time, the overall value of carbon emissions in the country is low and mostly the impact of the nodes is short. The Spring Festival is the most festive time of the year in China, during which people return to their hometowns in a mass migration of people (Jia et al., 2021). This mass movement forms the topology of a population movement network that connects most major cities, initiating the chain of transmission of COVID-19 in China (Govindan et al., 2020; Khan et al., 2020). However, the closure measures in most provinces make the node degree low and the temporal impact on carbon emissions short in extent.

**Figure 6:**
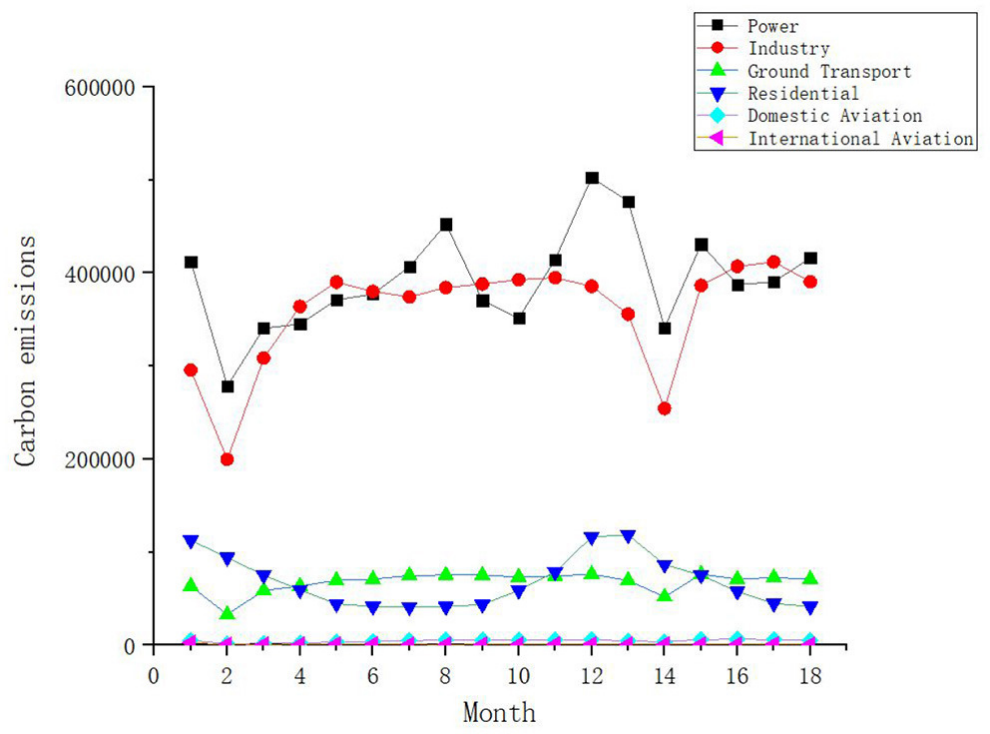
Trends in carbon emissions by sector over the period 2020.1–2021.6.

In the long term, 18 months of carbon emission data for each sector in China from 2020.01 to 2021.06 are selected, as shown in Figure 6. We can see that the impact of urban lockdowns on air pollution is short term, with rising emissions from energy, industry and land transport as closure policies continue to be liberalized across the region. It is worth noting that at month 14 (February 2020), greenhouse gas emissions from all sectors fall further to the level of the beginning of the year, due to the Spring Festival. The national promotion of "New Year in Place" has led to a reduction in transport and lower energy demand.

In addition, energy emissions increased significantly between Nov. 2020 and Mar. 2021, which can be explained by China's heating system. The impact of the blockade will be greater in the colder northern cities, which rely more on inefficient and inflexible coal-based winter central heating systems for residential and workplace buildings (Liu et al., 2021b). During the blockade, people are no longer required to work or go to school, so the heating of office/school buildings can be turned off completely, thereby reducing coal consumption. In contrast, the use of residential winter heating will not change much, as residential buildings must maintain their winter heating systems throughout the winter (Cui et al., 2021). Therefore, carbon emissions during this period are closely related to human behavior.

### 3.3. Carbon Emissions and Regional Heterogeneity

To further analyze carbon emissions from COVID-19 lockdown based on regional characteristics, we selected GDP per capita, total carbon emissions, passenger traffic, value added of the secondary industry in each province in 2020, and the number of confirmed cases from 2020.1.23 to 2020.3.8 (the period of COVID-19 lockdown in China) to construct a complex network visibility graph and further perform cluster analysis.

Specifically, the edges, mean path lengths, mean clustering coefficients and mean degrees of confirmed cases during the epidemic closure period were generated using the same method, and the best four clusters were selected by combining data on GDP per capita, total carbon emissions, passenger traffic and value added of secondary industries, as shown in Table 4. These two places have a high level of economic development, and at the same time focus on the development of tertiary industries rather than secondary industries, and therefore have relatively low carbon emissions. The provinces in Cluster 2 have a higher level of economic development and have higher carbon emissions, not only in terms of the development of the secondary sector but also in terms of the amount of passenger traffic. The provinces in the third cluster have a lower level of economic development compared to most of the first two clusters, and have a well-developed policy of epidemic lockdown, especially represented by the prevention and control in rural areas of Henan province. In addition, the geographical distribution is block-shaped. The provinces in the last cluster have an overall low level of carbon emissions, a better situation in the primary sector than in the previous clusters, and a relatively low level of economic development, but a strong industrial base.

**Table 4:** The optimal clustering by k-means.

**Cluster**	**Province**
1	Beijing, Shanghai Fujian, Guangdong, Hebei, Anhui, Hubei,
2	Hunan, Jiangxi, Jiangsu, Shandong, Sichuan, Zhejiang Chongqing, Guangxi, Guizhou, Hainan, Henan,
3	Inner Mongolia, Jilin, Qinghai, Shaanxi, Shanxi, Tianjin, Xinjiang, Yunnan Gansu, Heilongjiang,
4	Liaoning, Ningxia

From this we can conclude that (1) carbon emissions are closely related to the level of economic development and industrial structure. In the first-tier cities with a very high level of economic development, the secondary industry is not as well developed and emits less carbon. (2) Cities with a higher level of economic development also have relatively high carbon emissions, because they have the basic conditions for industrial development, so they are also divided into the same cluster. (3) Carbon emission levels during the epidemic are distributed in blocks on the ground. It is necessary to take measures from the aspects of industrial structure and transportation mode in combination with carbon trading in neighboring provinces.

## 4. Discussion

According to this study and the actual situation, firstly, after constructing the visible graph indicator, we found that the degree distribution is long-term correlated with the power-law distribution, that is, the most time nodes have a short range of influence. This shows that the lockdown helps maintain air quality (Barua and Nath, 2021). Changes in total carbon emissions of 30 provinces in 2020, COVID-19 brought short-term emission reductions, especially the impact on the transportation industry is significant (Thu et al., 2020). This is mainly due to the short period of change brought about by the lockdown. However, as the economy recovers, the future trajectory of "post-epidemic" CO2 emissions faces great uncertainty (Hanna et al., 2020). The economic downturn associated with COVID-19 will significantly increase the risk of resuming high-carbon, high-polluting economic activities in the future (Liu et al., 2021a). Therefore, in the post-economic era, reconciling economic recovery with carbon emission reduction is a new challenge for low-carbon economic development. Measures to reduce carbon emissions should be strengthened and stimulated, with a focus on rail transport infrastructure.

After further combining K-means analysis of indicators such as GDP per capita, passenger traffic and value-added of secondary industries in each province, we find that carbon emissions are closely related to the level of economic development and industrial structure of each province. In first-tier cities with very high levels of economic development have less developed secondary industries and lower carbon emissions. Cities with moderate economic development also have high carbon emissions, because they have the basic conditions for industrial development (Shao and Wang, 2021). Analysis of the data shows that industrial carbon emissions are the main industry in the country (Wang et al., 2021a). Furthermore, the level of carbon emissions during the pandemic is geographically blocky, especially for similar areas such as development at the economic level and industrial structure. Therefore, attention should be paid to carbon trading cooperation in neighboring provinces (Chapman and Tsuji, 2020), energy emission reduction should be coordinated with regional development and innovative development of low-carbon economy and green energy (Balsalobre-Lorente et al., 2020).

Specifically, with the spread of knowledge about the dangers of excessive greenhouse gas emissions, scholars around the world have shown great interest in reducing carbon emissions, developing a low-carbon economy and green energy. N. Absi et al. (2013) propose a multi-quota dynamic scheme for periodic carbon emission constraints in an attempt to achieve carbon emission limits through environmental constraints (Absi et al., 2013). Rui Sims et al. (2003) predict the impact of future technological developments on the cost of electricity generation and total carbon emissions, and suggest that the global power sector needs to improve the reduction of total carbon emissions through fuel switching, CO_2_ sequestration and greater use of renewable energy (Sims et al., 2003). It can be found that current research on reducing carbon emissions is focused on two main areas: reducing carbon emissions by improving carbon performance or shifting existing emission patterns (Rey-Hernández et al., 2020), or by finding new sources of energy using renewable energy (Iqbal et al., 2021; Khanna, 2021).

Finally, we take China's low-carbon economic measures during the COVID-19 pandemic as a reference to provide a new direction for energy conservation and emission reduction (Ionescu, 2021a). In China, to hedge against the impact of COVID-19 and the downward pressure on the economy, provinces across the country are expanding investment in infrastructure, creating a "new infrastructure" in the face of the epidemic (Gosens and Jotzo, 2020). The "new infrastructure" to hedge against the impact of COVID-19 will help transform China's energy structure and reduce CO2 emissions intensity in the following ways (Wang et al., 2020). Firstly, the accelerated construction of ultra-high voltage transmission lines will significantly improve power transmission capacity. Large-span and long-distance power transmission will effectively solve the consumption and output problems of new energy power generation. Secondly, the large-scale construction of new energy charging piles will effectively solve the bottleneck problem in the development of new energy vehicle industry and strongly support the rapid formation and development of China's large-scale electric vehicle market. Thirdly, the "new infrastructure" promotes low-carbon transportation (Zhang et al., 2021). The energy consumption of high-speed trains is much lower than that of aviation, road and high-speed rail transportation will reduce the overall energy consumption and carbon emission intensity of transport. The “new infrastructure” will help China achieve its goal of reaching peak CO_2_ emissions by around 2030, with some developed regions taking the lead in reaching the peak.

## 5. Conclusion

Based on complex network analysis and related methods, this paper explores the trend of carbon emissions and its internal logic in 30 Provinces of China during COVID-19. We subdivided the confirmed COVID-19 cases and carbon emissions into provincial daily data, making up for the lagging nature of data, low temporal frequency of data and non-dynamism in previous studies, and explored the characteristic causes of carbon emission changes. The heterogeneity among provinces was studied through K-means. Research shows that COVID-19 has a short time horizon for carbon emissions, and emissions are closely related to regional economic development level and industrial structure, with industrial emissions being the main industry in China. In addition, carbon emission levels are geographically contiguous, which suggests the need to focus on cooperation between neighboring provinces, optimize energy structures and promote the upgrading of “new infrastructure.”

The COVID-19 pandemic will not change the long-term trend in carbon emissions, but the unprecedented reduction in carbon emissions due to the lockdown is still worthy of further study. This study also has some limitations. In terms of research data, the number of cases during the lockdown period in China was only combined with some economic indicators, which can be further expanded in terms of covid-19-related indicators and economic indicators. Specifically, this paper suggests a direction for future research: while improved air quality is beneficial to human health, meteorological and climatic factors may be critical to the spread of COVID-19 (Sarwar et al., 2021). This suggests that mortality and morbidity data need to be collected to assess the impact of the measure on overall health. Secondly, the lockdown has important implications for work activities, travel and interpersonal constraints, including the impact on environmental quality. This requires a study of how human activity affects environmental quality, with human activity as the main focus (Marinello et al., 2021). These analyses are beyond the scope of our paper, but future research into these issues is necessary to understand their full implications. Overall, the research in this paper will help other governments make targeted policy decisions based on the COVID-19 pandemic, and draw valuable policy lessons from this unprecedented event while promoting low-carbon economic development.

## Data Availability Statement

The datasets presented in this study can be found in online repositories. The names of the repository/repositories and accession number(s) can be found in the article/Supplementary Material.

## Author Contributions

JH and JC conceived the research. PZ and HL designed the analyses and compiled the data. SH and MW conducted the analyses. SH, MW, and NL wrote the paper. All authors read and approved the final manuscript.

## Funding

This study was funded by Science and Technology Innovation 2030 New Generation Artificial Intelligence, Major Project (Grant no. 2020AAA0107704), the Scientific Research Fund Project of Yunnan Education Department (Grant no. 2021J0586), and the National Natural Science Foundation of China (Grant nos. 72074237 and 62073263), Technology-Scientific and Technological Innovation Team of Shaanxi Province (Grant no. 2020TD-013).

## Conflict of Interest

The authors declare that the research was conducted in the absence of any commercial or financial relationships that could be construed as a potential conflict of interest.

## Publisher's Note

All claims expressed in this article are solely those of the authors and do not necessarily represent those of their affiliated organizations, or those of the publisher, the editors and the reviewers. Any product that may be evaluated in this article, or claim that may be made by its manufacturer, is not guaranteed or endorsed by the publisher.
