# Supplementary figures and images for: Difference and Cluster Analysis on the Carbon Dioxide Emissions in China During COVID-19 Lockdown via a Complex Network Model
Source: Front Psychol. 2022 Jan 12;12:795142. doi: 10.3389/fpsyg.2021.795142 (PMC8790068; doi:10.3389/fpsyg.2021.795142)

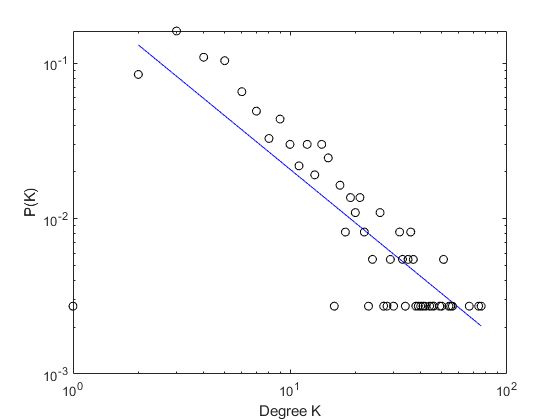

Supplement: Supplementary file 4 [file Data_Sheet_1.ZIP › 1.jpg]

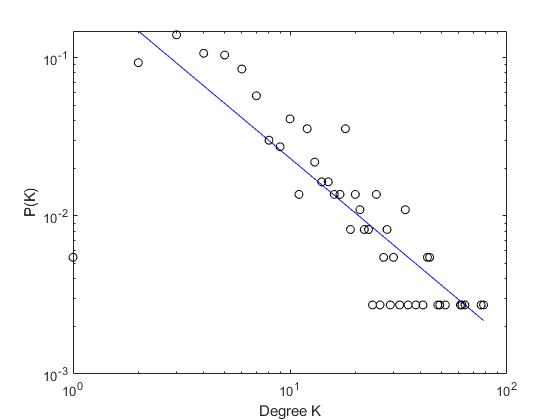

Supplement: Supplementary file 4 [file Data_Sheet_1.ZIP › 2.jpg]

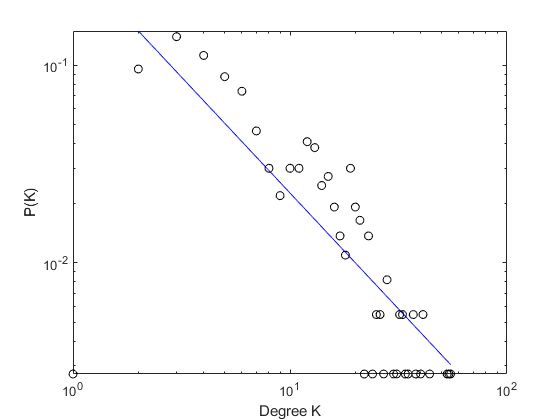

Supplement: Supplementary file 4 [file Data_Sheet_1.ZIP › 3.jpg]

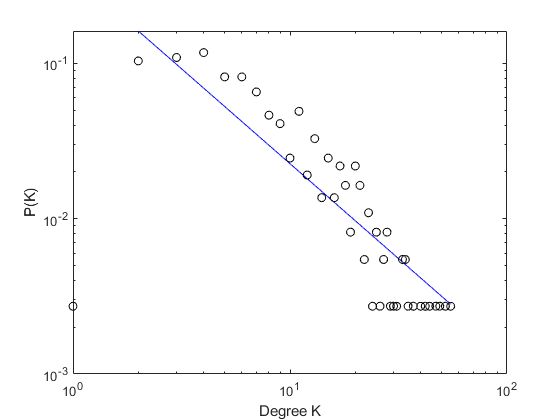

Supplement: Supplementary file 4 [file Data_Sheet_1.ZIP › 4.jpg]

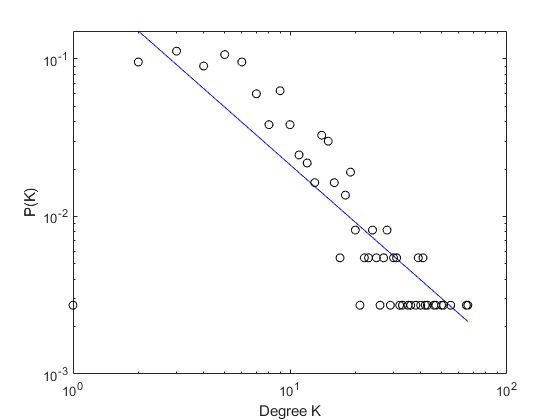

Supplement: Supplementary file 4 [file Data_Sheet_1.ZIP › 5.jpg]

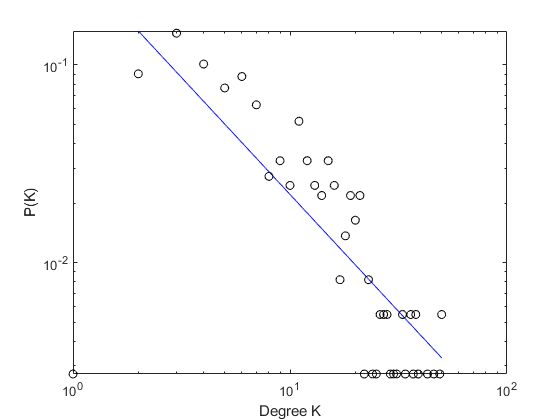

Supplement: Supplementary file 4 [file Data_Sheet_1.ZIP › 6.jpg]

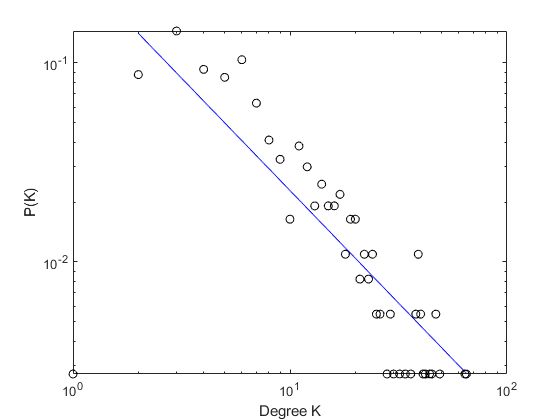

Supplement: Supplementary file 4 [file Data_Sheet_1.ZIP › 7.jpg]

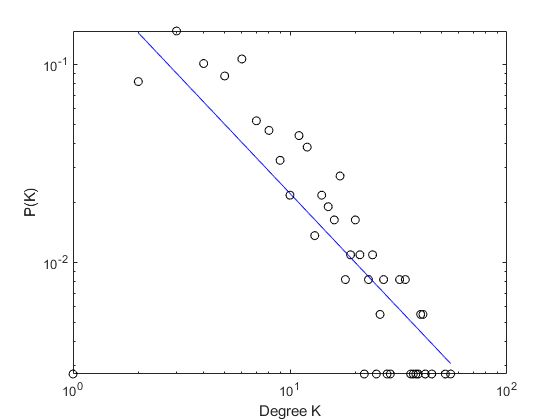

Supplement: Supplementary file 4 [file Data_Sheet_1.ZIP › 8.jpg]

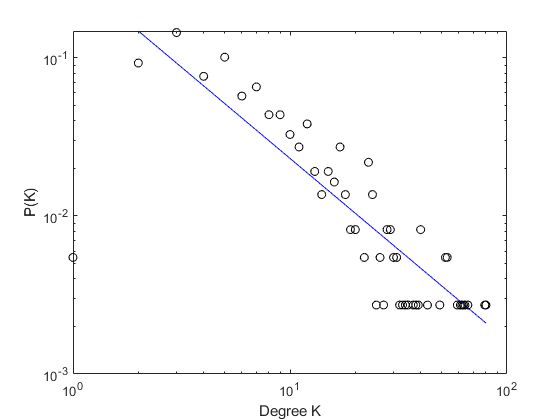

Supplement: Supplementary file 4 [file Data_Sheet_1.ZIP › 9.jpg]

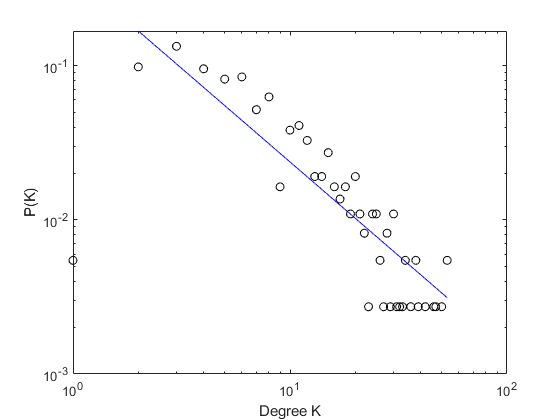

Supplement: Supplementary file 4 [file Data_Sheet_1.ZIP › 10.jpg]

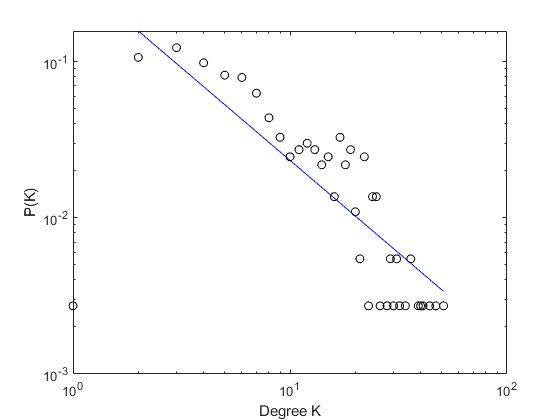

Supplement: Supplementary file 4 [file Data_Sheet_1.ZIP › 11.jpg]

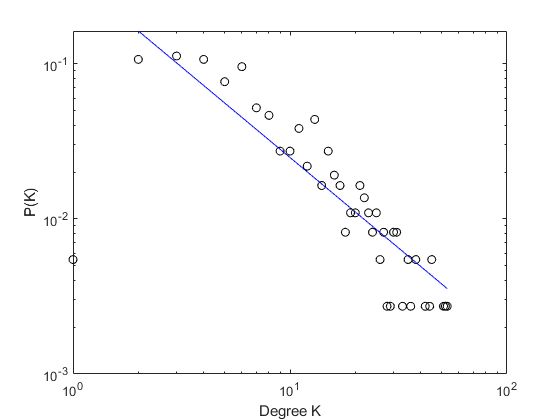

Supplement: Supplementary file 4 [file Data_Sheet_1.ZIP › 12.jpg]

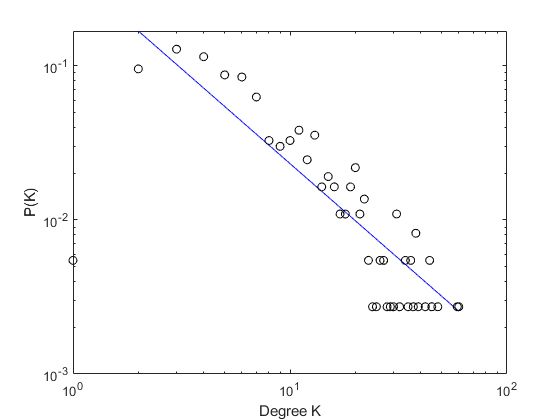

Supplement: Supplementary file 4 [file Data_Sheet_1.ZIP › 13.jpg]

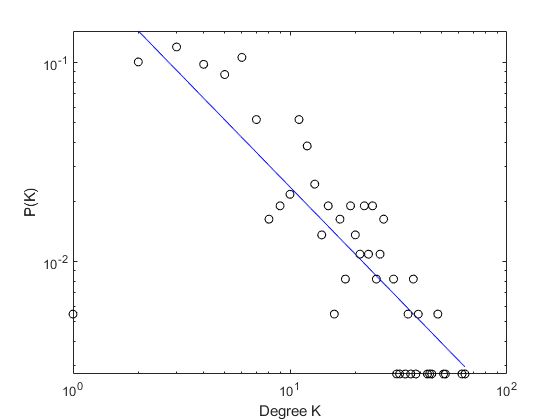

Supplement: Supplementary file 4 [file Data_Sheet_1.ZIP › 14.jpg]

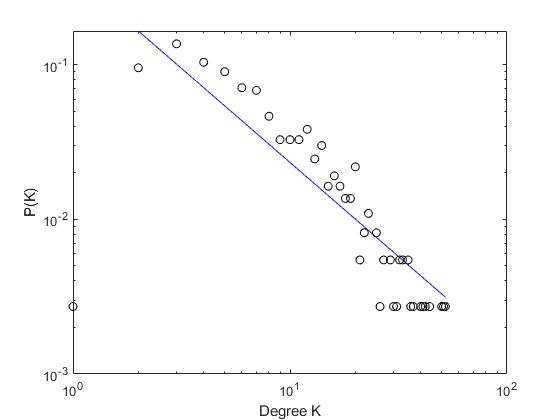

Supplement: Supplementary file 4 [file Data_Sheet_1.ZIP › 15.jpg]

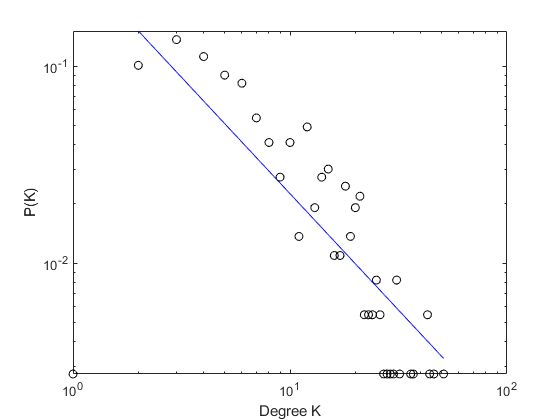

Supplement: Supplementary file 4 [file Data_Sheet_1.ZIP › 16.jpg]

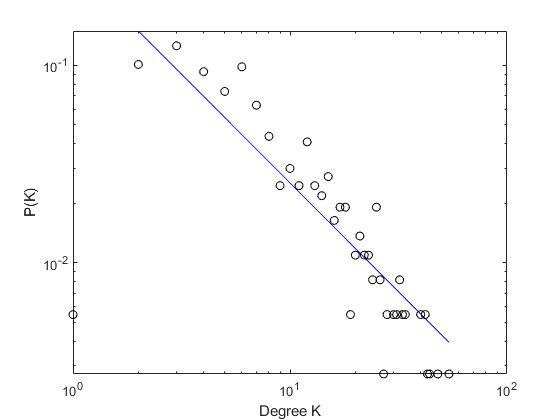

Supplement: Supplementary file 4 [file Data_Sheet_1.ZIP › 17.jpg]

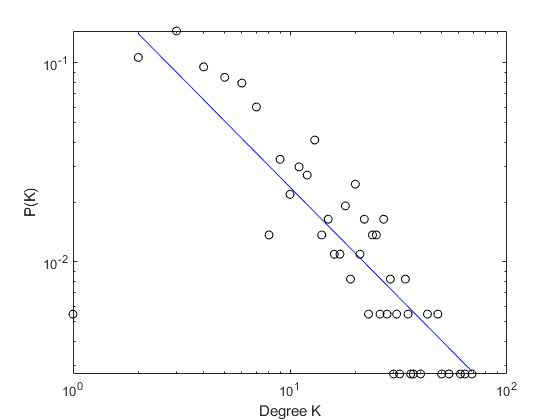

Supplement: Supplementary file 4 [file Data_Sheet_1.ZIP › 18.jpg]

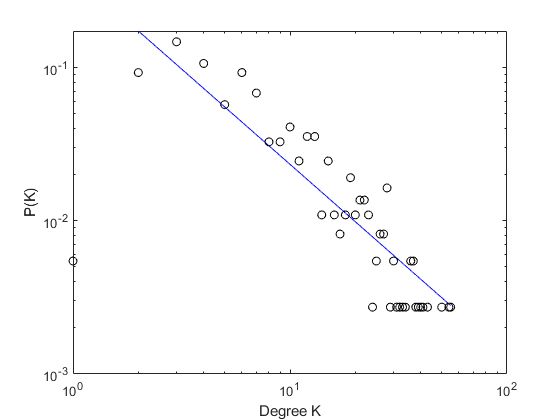

Supplement: Supplementary file 4 [file Data_Sheet_1.ZIP › 19.jpg]

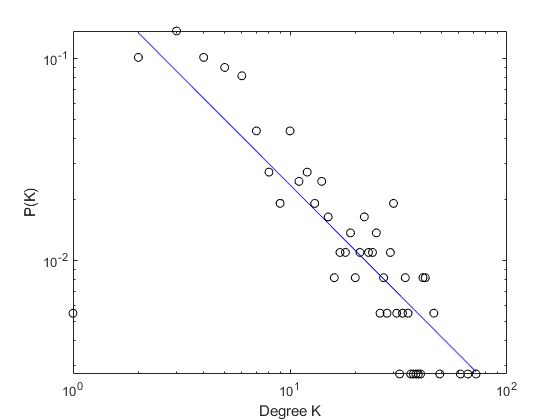

Supplement: Supplementary file 4 [file Data_Sheet_1.ZIP › 20.jpg]

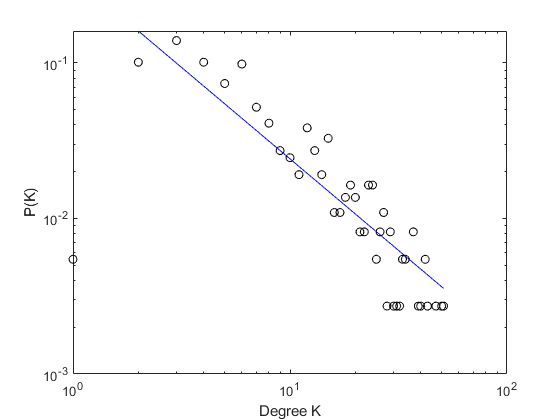

Supplement: Supplementary file 4 [file Data_Sheet_1.ZIP › 21.jpg]

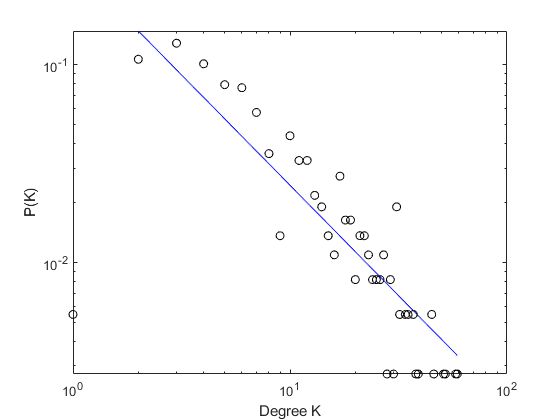

Supplement: Supplementary file 4 [file Data_Sheet_1.ZIP › 22.jpg]

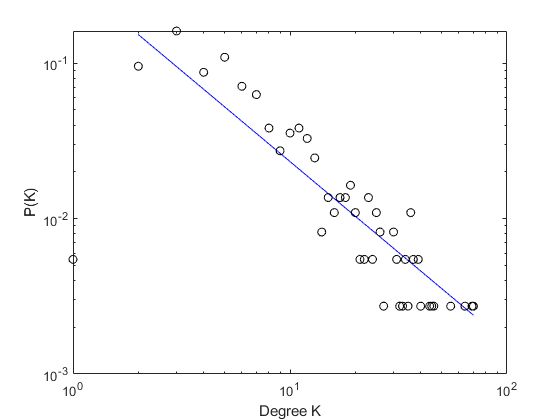

Supplement: Supplementary file 4 [file Data_Sheet_1.ZIP › 23.jpg]

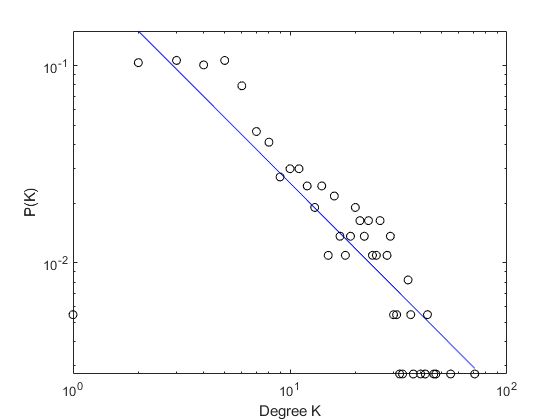

Supplement: Supplementary file 4 [file Data_Sheet_1.ZIP › 24.jpg]

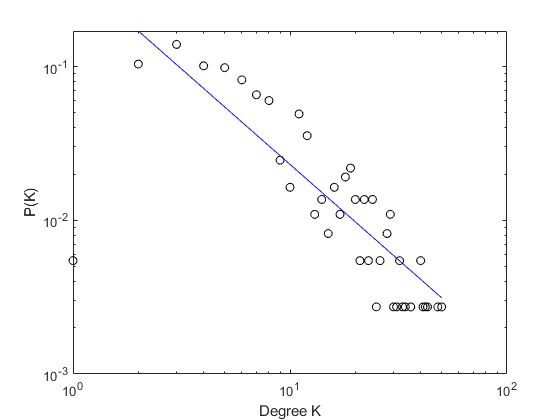

Supplement: Supplementary file 4 [file Data_Sheet_1.ZIP › 25.jpg]

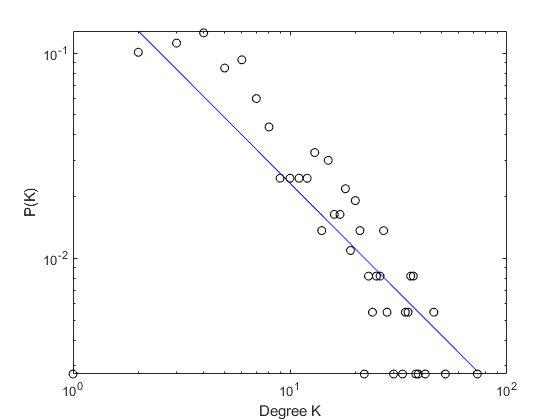

Supplement: Supplementary file 4 [file Data_Sheet_1.ZIP › 27.jpg]

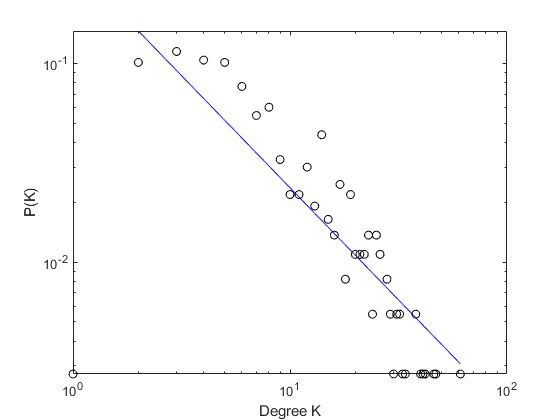

Supplement: Supplementary file 4 [file Data_Sheet_1.ZIP › 28.jpg]

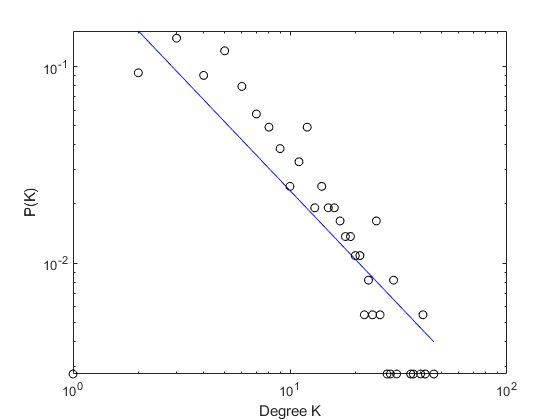

Supplement: Supplementary file 4 [file Data_Sheet_1.ZIP › 29.jpg]

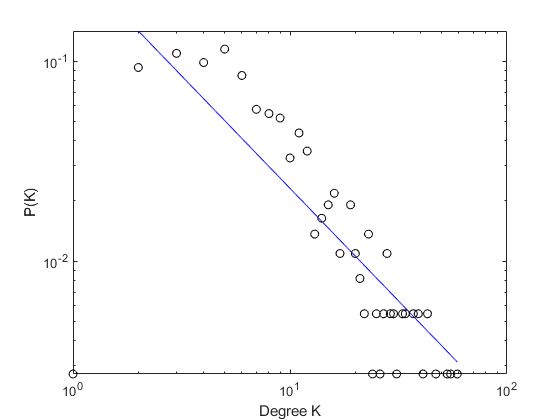

Supplement: Supplementary file 4 [file Data_Sheet_1.ZIP › 30.jpg]

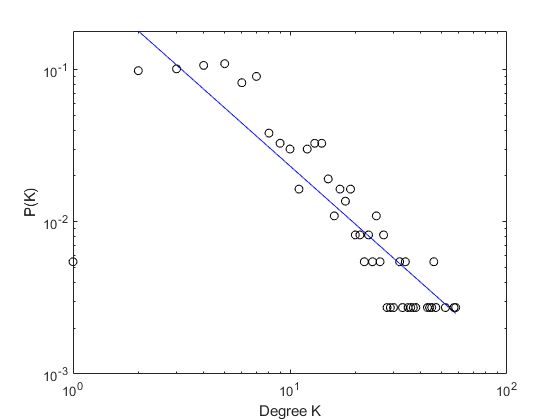

Supplement: Supplementary file 4 [file Data_Sheet_1.ZIP › 31.jpg]

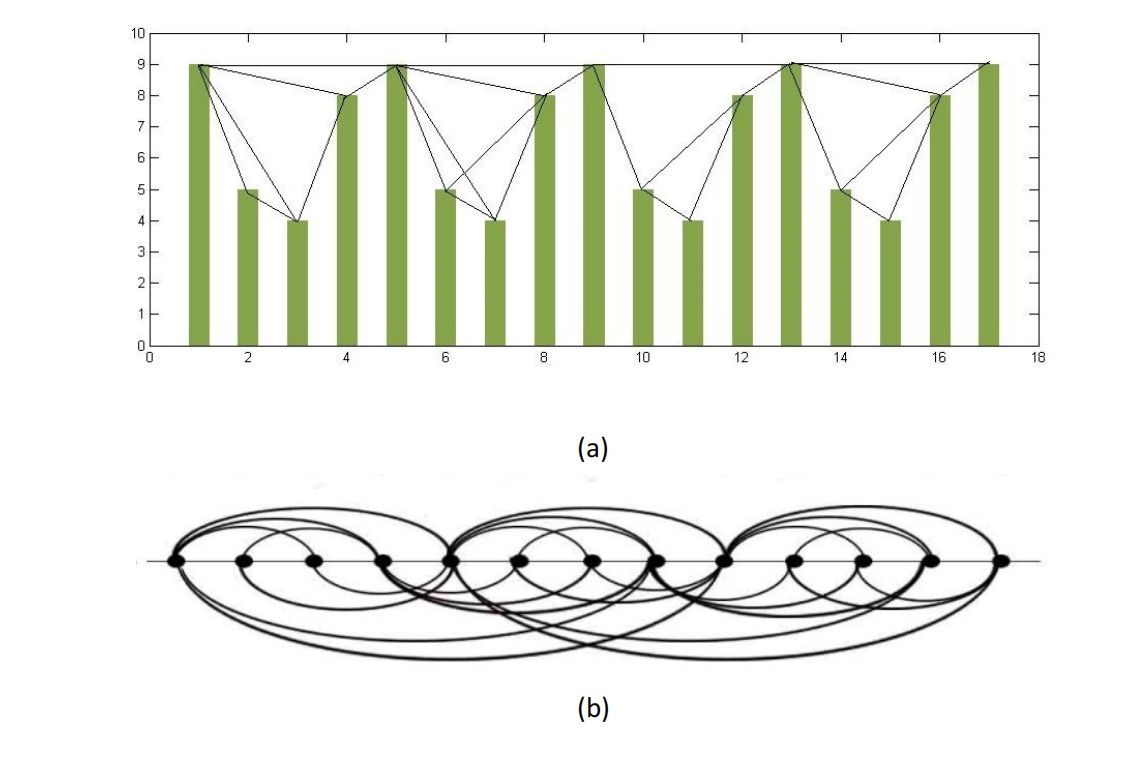

Supplement: Supplementary file 4 [file Data_Sheet_1.ZIP › image2.jpg]

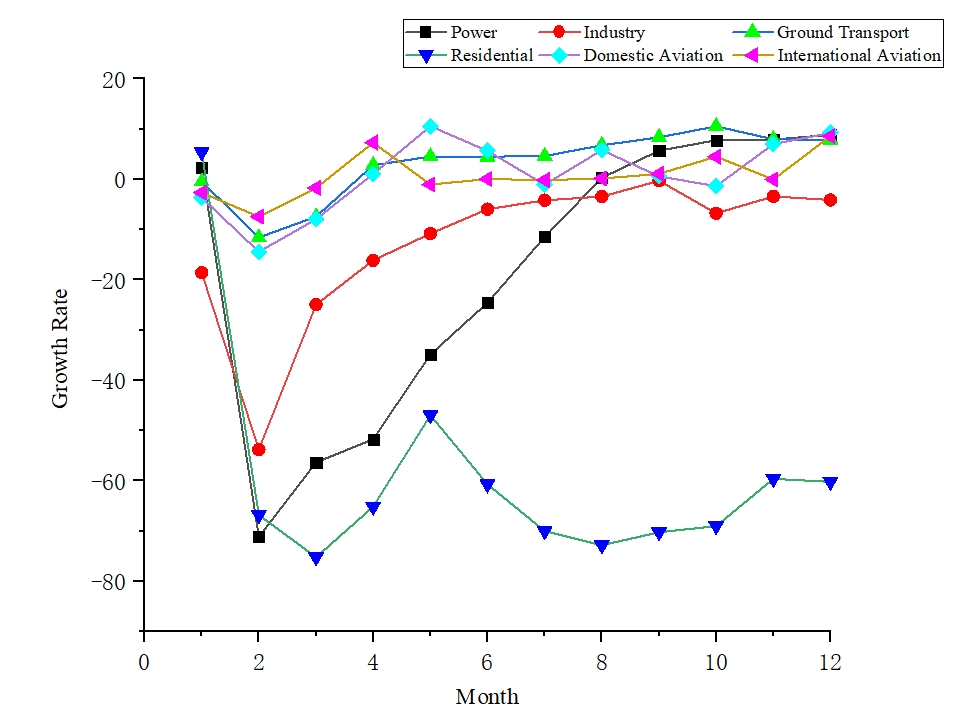

Supplement: Supplementary file 4 [file Data_Sheet_1.ZIP › trend line.jpg]

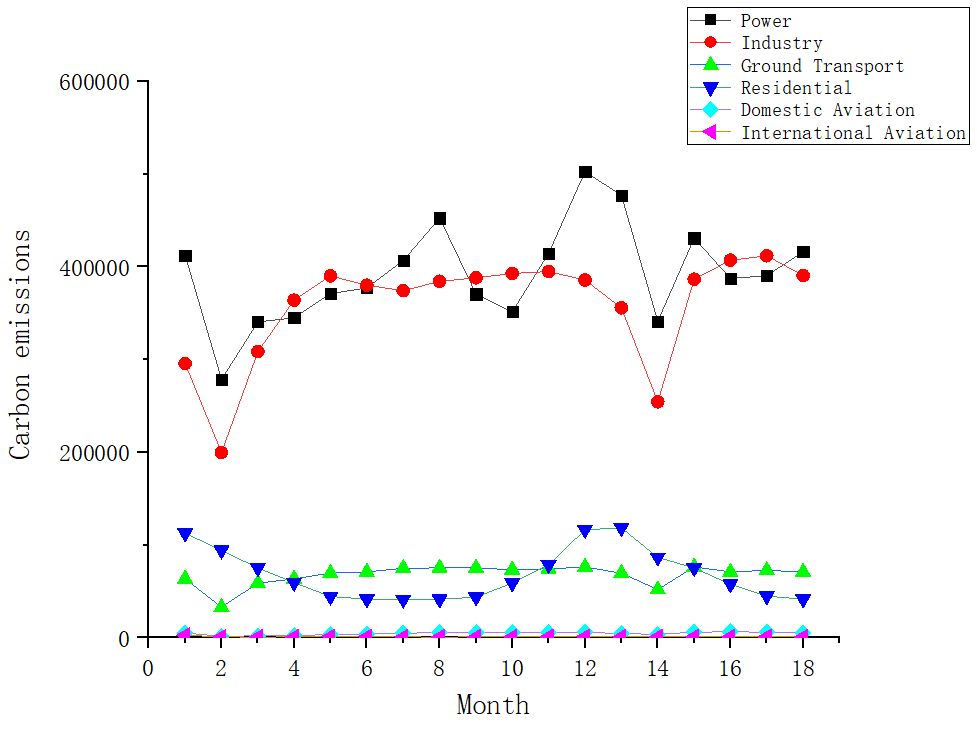

Supplement: Supplementary file 4 [file Data_Sheet_1.ZIP › trends.jpg]

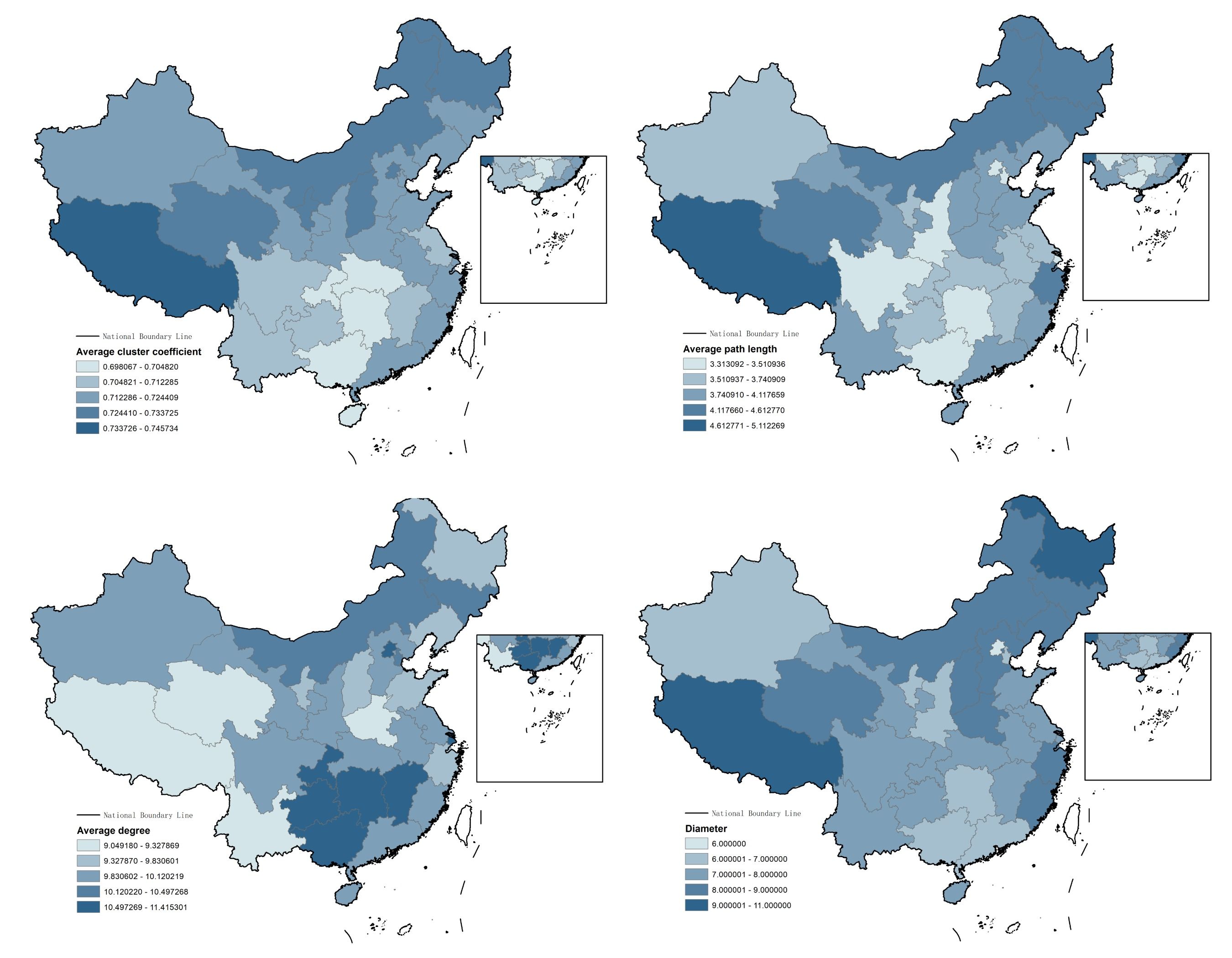

Supplement: Supplementary file 4 [file Data_Sheet_1.ZIP › ZB.jpg]

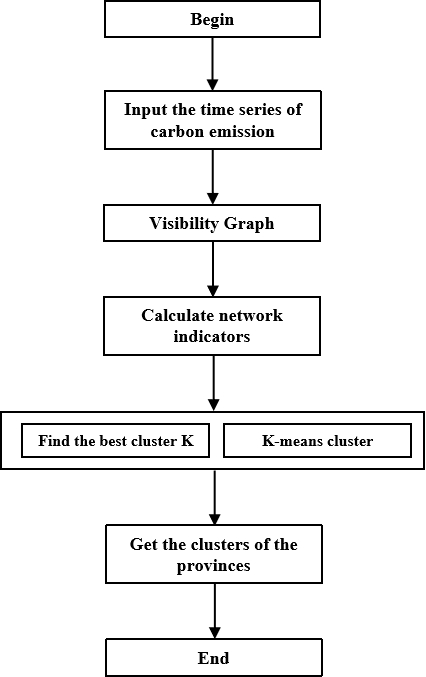

Supplement: Supplementary file 4 [file Data_Sheet_1.ZIP › flowchart.jpg]
